# The Efficacy of Vortioxetine on Anhedonia in Patients With Major Depressive Disorder

**DOI:** 10.3389/fpsyt.2019.00017

**Published:** 2019-01-31

**Authors:** Bing Cao, Caroline Park, Mehala Subramaniapillai, Yena Lee, Michelle Iacobucci, Rodrigo B. Mansur, Hannah Zuckerman, Lee Phan, Roger S. McIntyre

**Affiliations:** ^1^School of Public Health, Peking University, Beijing, China; ^2^Mood Disorders Psychopharmacology Unit, Toronto Western Hospital, University Health Network, Toronto, ON, Canada; ^3^Faculty of Medicine, Institute of Medical Science, University of Toronto, Toronto, ON, Canada; ^4^Brain and Cognition Discovery Foundation, Toronto, ON, Canada; ^5^Department of Psychiatry, University of Toronto, Toronto, ON, Canada; ^6^Department of Pharmacology, University of Toronto, Toronto, ON, Canada

**Keywords:** major depressive disorder, anhedonia, function, vortioxetine, quality of life, antidepressants

## Abstract

**Background:** Anhedonia is a common, persistent, and disabling phenomenon in treated adults with Major Depressive Disorder (MDD). Hitherto, relatively few antidepressant agents have been evaluated with respect to their effect on anhedonia in MDD.

**Methods:** This is a *post-hoc* analysis of a primary study that sought to evaluate the sensitivity to change of the THINC-integrated tool (THINC-it) in MDD (ClinicalTrials.gov Identifier: NCT03053362). Adults meeting DSM-5 criteria for MDD with at least moderate depressive symptom severity [i.e., Montgomery Åsberg Depression Rating Scale (MADRS) total score ≥20] were eligible. Subjects were recruited between October 2017 and August 2018 in Toronto, Ontario at the Brain and Cognition Discovery Foundation. All subjects received open-label vortioxetine (10–20 mg/day, flexibly-dosed) for 8 weeks. Herein, the primary outcome of interest was the change from baseline to endpoint in the Snaith-Hamilton Pleasure Scale (SHAPS) total score, as well as the MADRS anhedonia factor. The mediational effects of improvements in anhedonia on general function and quality of life, as measured by the Sheehan Disability Scale (SDS) and the 5-Item World Health Organization Well-Being Index (WHO-5), were secondarily assessed.

**Results:** A total of 100 subjects with MDD were enrolled in the primary study and began treatment with vortioxetine. Vortioxetine significantly improved anhedonia as evidenced by significant baseline to endpoint improvements in SHAPS and MADRS anhedonia factor scores (*p* < 0.0001). Improvements in the SHAPS and the MADRS anhedonia factor correlated with improvements in general function (i.e., SDS) and quality of life (i.e., WHO-5) (*p* < 0.0001). Notably, improvements in anhedonia were found to mediate the association between improvements in overall depressive symptom severity (i.e., MADRS total score) and social functioning (i.e., social life component of the SDS) (*p* = 0.026).

**Conclusion:** The unmet need in depression is to improve patient functioning and other patient-reported outcomes (e.g., quality of life). Antidepressant interventions capable of attenuating anhedonia as well as cognitive dysfunction in MDD may help in this regard, as improvement in these domains have been associated with improvement in psychosocial function and quality of life.

## Introduction

Major depressive disorder (MDD) is a leading cause of disability worldwide and is associated with significant economic burden (1). Approximately 50% of the illness burden and costs attributable to MDD is due to impairments in function (e.g., impaired workplace function, short-/long-term disability) (2, 3). Replicated evidence indicate that disturbances in motivation (and cognition) are persisting deficits in MDD and mediate poor functional outcomes in MDD.

Anhedonia is defined as an impaired capacity to experience or anticipate pleasure (4). According to the Diagnostic and Statistical Manual of Mental Disorders, fifth edition (DSM-5), anhedonia and depressed mood are among the key diagnostic features that characterize a major depressive episode (MDE) as part of MDD (5). Notably, anhedonia has been associated with disturbances of central dopaminergic, mesolimbic, and mesocortical reward circuit pathways, which involve brain regions such as the ventral tegmental area (VTA), ventral striatum, and pre-frontal cortex (6).

Anhedonia is a common symptom of MDD, and is reported in ~75% of patients (7). Importantly, anhedonia and impaired reward learning have been associated with poorer disease prognosis and suboptimal treatment response (8). In addition to being a common symptom of MDD, it is often a persisting dimension amongst individuals with MDD receiving disparate treatments (9). The hazards posed by anhedonia, as well as the suboptimal anti-anhedonia effects of many available antidepressants, provides the impetus for specifically evaluating the efficacy of newer treatments on this dimension. Preliminary evidence suggests, for example, that agomelatine and ketamine may exert clinically relevant effects on measures of anhedonia (10, 11).

Vortioxetine is a multimodal antidepressant with multiple effector neurotransmitter systems, including serotonin (5-HT), norepinephrine (NE), dopamine, amino acids, histamine (HA), and cholinergic systems (12–15). Results from meta- and network analyses indicate that vortioxetine is generally well-tolerated and efficacious at reducing MDD illness severity (16–18). Moreover, in 2018, the product insert for vortioxetine in the USA was updated to include mention of vortioxetine's independent pro-cognitive effects in MDD.

The pharmacodynamic profile of vortioxetine, as well as the pro-cognitive effects of this agent (13), provide the basis for hypothesizing that vortioxetine may be able to attenuate measures of anhedonia in adults with MDD. In addition, no previous study has evaluated the anti-anhedonia effects of vortioxetine, and since anhedonia (like cognition) has been shown to be an important mediator of overall clinical improvement in MDD. Herein, we sought to determine whether vortioxetine improved measures of anhedonia and to what extent improvements in anhedonia correlate with overall function and quality of life.

## Methods

### Study Population

This is a *post-hoc* analysis of a primary study that sought to evaluate the sensitivity to change of the THINC-integrated tool (THINC-it) in MDD (ClinicalTrials.gov Identifier: NCT03053362). Adults meeting DSM-5 criteria for MDD with at least moderate depressive symptom severity [i.e., Montgomery Åsberg Depression Rating Scale (MADRS) total score ≥20] were eligible. Subjects were recruited between October 2017 and August 2018 in Toronto, Ontario at the Brain and Cognition Discovery Foundation. All subjects received open-label vortioxetine (10–20 mg/day, flexibly-dosed) for 8 weeks. Ninety-five female and male patients with DSM-5-defined MDD between the ages of 18 and 65 were included in the analysis. Approval from a local Institutional Review Board was obtained prior to initiating the study and all eligible participants provided written informed consent.

### Eligibility Criteria

Patients who met the following eligibility criteria were included into the study: (1) provided written informed consent, (2) male or female between 18 and 65 years of age, (3) current diagnosis of a major depressive episode (MDE) as part of MDD as per DSM-5 criteria, (4) current MDE was confirmed by the Mini International Neuropsychiatric Interview (M.I.N.I 5.0.), (5) outpatient of a psychiatric setting, (6) MADRS score ≥20 at screening and baseline, (7) history of at least one prior MDE formally diagnosed by a healthcare provider or validated by previous treatment (e.g., guideline-informed pharmacotherapy and/or manual-based psychotherapy).

The exclusion criteria were: (1) current alcohol and/or substance use disorder as confirmed by the M.I.N.I 5.0, (2) presence of a comorbid psychiatric disorder that was a focus of clinical concern (3) medications approved and/or employed off-label for cognitive dysfunction (e.g., psychostimulants), (4) medications for a general medical disorder that, in the opinion of the investigator, could affect cognitive function, (5) use of benzodiazepines within 12 h of cognitive assessments, (6) consumption of alcohol within 8 h of cognitive assessments, (7) inconsistent use or abuse of marijuana, (8) physical, cognitive, or language impairments sufficient to adversely affect data derived from cognitive assessments, (9) diagnosed reading disability or dyslexia, (10) clinically significant learning disorder by history, (11) electroconvulsive therapy (ECT) in the last 6 months, (12) history of moderate or severe head trauma (e.g., loss of consciousness for >1 h), other neurological disorders, or unstable systemic medical diseases that, in the opinion of the investigator, are likely to affect the central nervous system, (13) pregnant and/or breastfeeding, (14) received investigational agents as part of a separate study within 30 days of the screening visit, (15) actively suicidal or evaluated as being at high suicide risk as per clinical judgment using the Columbia-Suicide Severity Rating Scale, (16) currently receiving treatment with Monoamine Oxidase Inhibitors (MAOIs), antibiotics such as linezolid, or intravenous methylene blue, (17) previous hypersensitivity reaction to vortioxetine or any components of the formulation.

### Study Procedure

Patients taking other medications for depression were tapered off these drugs as instructed by the treating clinician (the tapering period was based on the time it takes for vortioxetine to reach therapeutically significant plasma levels in the bloodstream). Subjects with MDD were dosed with vortioxetine (10–20 mg, flexibly dosed) daily for 8 weeks. All enrolled participants will receive vortioxetine 10 mg/day for the first 2 weeks. They also had the option of moving up to 20 mg; however, dose adjustment was based on tolerability and clinical response as assessed by the treating clinician. Subjects underwent five visits (screening, week 0: baseline, week 2, week 4, and week 8: endpoint). The MADRS total score was evaluated at all five visits, and the SHAPS and SDS scores were evaluated at three different time points (i.e., week 0, week 2, and week 8).

### Outcome Measures

The primary outcome of the analysis was change in anhedonia, as measured by the baseline to endpoint change in Snaith–Hamilton Pleasure Scale (SHAPS) and the MADRS anhedonia factor [i.e., which was based on items 1 (apparent sadness), 2 (reported sadness), 6 (concentration difficulties), 7 (lassitude), 8 (inability to feel)]. Relevant secondary measures were functional impairment, as measured by the Sheehan Disability Scale (SDS), which is a brief self-report measure that evaluates three functional domains (i.e., work/school, social life, and family life or home responsibilities); and quality of life, as measured by the 5-Item World Health Organization Well-Being Index (WHO-5), which is a five-item self-report measure assessing subjective psychological well-being. For the logistic regression analyses, clinical response for anhedonia was defined as having a SHAPS score improvement of ≥50% from baseline, and remission of anhedonia was defined as having a follow-up SHAPS score of ≤ 3 (19).

### Statistical Analysis

Continuous variables were summarized as means and standard deviations (SDs) or medians and interquartile ranges (IQRs). Categorical variables were summarized as frequencies and proportions. The repeated measures mixed model analysis was used to evaluate changes in the SHAPS and MADRS scores. A multivariate logistic regression model was used to analyze differences in demographic and clinical variables (i.e., sex; age; total years of education; current use of alcohol, nicotine, marijuana; age of MDD onset, length of current MDE; family history of mental illness) that might affect anhedonia in patients with MDD. Odds ratios (ORs) and their 95% confidence intervals (CIs) were estimated using maximum likelihood methods. Correlations among different scale scores (i.e., MADRS, SHAPS, SDS, WHO-5) were calculated using the Pearson correlation coefficient. Mediational analysis was used to estimate indirect effects. Herein, a mediation analysis was used to determine the extent to which the association between changes in depressive symptom severity (ΔMADRS), changes in functional impairment (ΔSDS), and changes in well-being (ΔWHO-5) were mediated by improvements in anhedonia (ΔSHAPS) in patients treated with vortioxetine over 8 weeks. Significance was set to *p* < 0.05, two-sided. All statistical analyses were conducted using SPSS software, version 22.0.

## Results

### Characteristics of Patients With MDD at Baseline

One hundred and forty-four subjects with MDD provided written, informed consent, and underwent screening. Of 95 eligible subjects from the primary study, 92 (96.8%) and 79 (83.2%) completed the week 2 visit and week 8 visit, respectively. The mean age of subjects at baseline was 38.9 years (SD = 12.9), and 62 (65.3%) subjects were female. The median age of MDD onset was 16.0 years (IQR = 13.0–25.0), and the median length of current MDE duration was 8 months (IQR = 5.0–25.0). Sixty-one subjects (64.8%) reported a positive family history of mental illness. With respect to clinical characteristics, the mean (SD) MADRS and SDS of subjects at baseline were 32.3 (7.3) and 20.9 (6.0), respectively. The characteristics of MDD patients at baseline are described in Table 1.

**Table 1:** Characteristics of patients with MDD at baseline.

**Variables**	**Baseline clinical characteristics of patients (*n* = 95)**
**CATEGORICAL VARIABLES**	
Sex (female/male); *n* (%)	62/33 (65.3/34.7)
Current alcohol use (at least weekly) (yes/no); *n* (%)	32/63 (33.7/66.3)
Current Nicotine use (yes/no); *n* (%)	22/73 (31.6.2/68.4)
Current Marijuana use (yes/no); *n* (%)	29/66 (30.5/69.5)
Family history of mental illness (yes/no); *n* (%)	61/34 (64.2/35.8)
Presence of psychiatric comorbidity (yes/no); *n* (%)	49/45 (51.6/48.4)
Presence of general comorbidity (yes/no); *n* (%)	59/36 (62.1/37.9)
**CONTINUOUS VARIABLES**	
Age in years; mean (SD)	38.9 (12.9)
BMI (kg/m^2^); mean (SD)	28.5 (6.5)
Total years of education; mean (SD)	15.7 (3.1)
Age of MDD onset in years; median (IQR)	16.0 (13.0, 25.0)
Number of lifetime episodes; median (IQR)	5.0 (3.0, 20.0)
Duration of illness in years; mean (SD)	15.0 (8.0, 26.0)
Length of Current MDE in months; median (IQR)	8.0 (5.0, 24.0)
Number of current psychiatric medications; median (IQR)	1.0 (0.0, 2.0)
Number of past psychiatric medications; median (IQR)	1.0 (0.0, 2.0)
MADRS total score; mean (SD)	32.3 (7.3)
SDS total score; mean (SD)	20.9 (6.0)
SDS work; mean (SD)	6.9 (2.9)
SDS social; mean (SD)	7.2 (2.3)
SDS family life; mean (SD)	7.1 (2.2)
WHO-5; mean (SD)	14.8 (11.3)

*MDE, major depressive episode; MADRS, Montgomery Åsberg Depression Rating Scale; SHAPS, Snaith-Hamilton Pleasure Scale; SDS, Sheehan Disability Scale; WHO-5, The World Health Organization- Five Well-Being Index*.

### Efficacy of Vortioxetine on Anhedonia Outcomes

**Figure 1:**
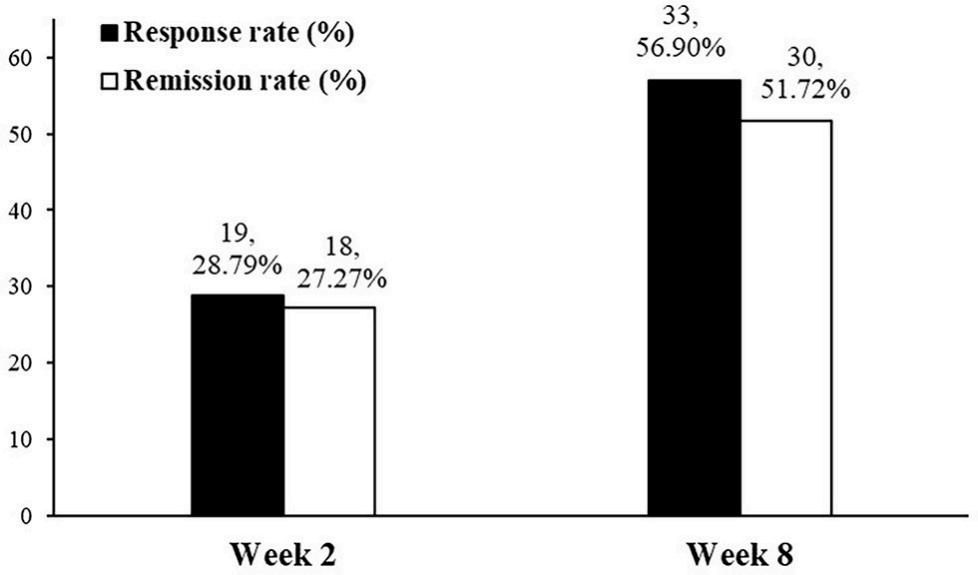
Response and remission rate of anhedonia in patients with MDD using SHAPS at weeks 2 and 8.

Treatment with vortioxetine significantly improved measures of anhedonia between baseline and endpoint (i.e., week 8) (ΔSHAPS = −2.9, 95% CI: −3.7, −2.2, *z* = −7.88, *p* < 0.0001; ΔMADRS anhedonia factors = −7.1, 95% CI: −8.2, −5.9, *z* = −11.90, *p* < 0.0001). Response and remission rates at endpoint were 56.9 and 51.72%, respectively (Figure 1). Significant correlations were found between the SHAPS and MADRS anhedonia factor at baseline (*r* = 0.474, *p* < 0.0001), week 2 (*r* = 0.669, *p* < 0.0001), and week 8 (*r* = 0.474, *p* < 0.0001).

Repeated measures mixed model analyses were used to assess for changes in anhedonia after 2 and 8 weeks of treatment with vortioxetine when compared to baseline (Table 2). Among the subjects who completed the 2 weeks follow-up, 71.7% (66/92) had SHAPS >3 at baseline (i.e., the presence of clinically significant anhedonia). Between baseline and week 2, these subjects exhibited significant improvements in anhedonia, as indicated by both the change in SHAPS score (ΔSHAPS = −1.0, 95% CI: −1.7, −0.3, *z* = −2.88, *p* = 0.004) and the change in MADRS anhedonia factor (ΔMADRS anhedonia factor = −3.8, 95% CI: −4.9, −2.7, *z* = −7.22, *p* < 0.0001). Anhedonia response and remission rates for the 66 MDD patients with anhedonia at week 2 were 28.79 and 27.27%, respectively (Figure 1). Among the subjects who completed the 8 weeks follow-up, 73.4% (58/79) had SHAPS >3 at baseline. Furthermore, among the subjects withdrew, 70.6% (11/16) had SHAPS >3 at baseline. No significant difference was observed between the patients completed the follow-up and withdrew. Both the changes of SHAPS and MADRS anhedonia factor scores have significant improvement between baseline and endpoint (ΔSHAPS = −2.9, 95% CI: −3.7, −2.2, *z* = –7.88, *p* < 0.0001; ΔMADRS anhedonia factors = −7.1, 95% CI: −8.2, −5.9, *z* = −11.90, *p* < 0.0001). The anhedonia response and remission rate were reached 56.9 and 51.72%, respectively (Figure 1). In addition, the changes of SHAPS and MADRS anhedonia factor scores with treatment of vortioxetine in the subgroup of patients with anhedonia (i.e., SHAPS score >3 at baseline) were shown in Supplemental Table 1. The changes in the subgroup was similar as overall patient both in weeks 2 and 8.

**Table 2:** The changes of SHAPS and MADRS anhedonia factor scores with treatment of vortioxetine.

**Variables**	**Baseline (*n* = 95)**	**Week 2 (*n* = 92)**				**Week 8 (*n* = 79)**			
	**M ± SD**	**M ± SD**	***Δbaseline (95% CI)***	***z***	***p***	**M ± SD**	***Δbaseline (95% CI*)****	***z***	***p***
SHAPS score	6.2 ± 3.8	5.2 ± 4.5	−1.0 (−1.7, −0.3)	−2.88	0.004^*^	3.3 ± 3.8	−2.9 (−3.7, −2.2)	−7.88	< 0.0001^*^
MADRS anhedonia factor	18.3 ± 3.7	14.5 ± 5.1	−3.8 (−4.9, −2.7)	−7.22	< 0.0001^*^	11.4 ± 6.1	−7.1 (−8.2, −5.9)	−11.90	< 0.0001^*^

* *Indicates significant differences from baseline*.

The logistic regression analysis indicated that none of the analyzed demographic and clinical variables significantly influenced SHAPS-defined anhedonia at baseline. However, the results demonstrated that gender was predictive of remission at endpoint (i.e., females AOR = 0.139, 95% CI: 0.024, 0.802, *p* = 0.027). Moreover, current marijuana use (AOR = 6.056, 95% CI: 1.168, 31.397, *p* = 0.032), older age of MDD onset (AOR = 1.086, 95% CI: 1.009, 1.169, *p* = 0.028), and having a family history of mental illness (AOR = 5.476, 95%CI: 1.156, 25.947, *p* = 0.032) increased the probability of non-remission at endpoint (Table 3).

**Table 3:** Logistic regression of the factors affecting anhedonia in patients with MDD.

**Factors**	**AOR**	**95%CI**	***p-values***
**TOTAL PATIENTS (*N* = 95): WITHOUT ANHEDONIA VS. WITH**			
**ANHEDONIA AT BASELINE**			
Age (years)	1.013	0.971, 1.056	0.550
Sex (male/female)	1.165	0.443, 3.059	0.757
Total years of education (years)	1.027	0.875, 1.205	0.745
Current alcohol use (at least weekly) (yes/no)	0.504	0.196, 1.299	0.156
Current Nicotine use (yes/no)	2.141	0.588, 7.802	0.248
Current Marijuana use (yes/no)	0.648	0.237, 1.774	0.399
Age of MDD onset (years)	0.986	0.941, 1.032	0.539
Length of Current MDE (months)	1.008	0.993, 1.024	0.297
Family history of mental illness (yes/no)	0.762	0.276, 2.104	0.601
PATIENTS WITH ANHEDONIA^*^ (*N* = 58): REMITTER VS. NON-REMITTER			
**AT ENDPOINT**			
Age (years)	1.022	0.968, 1.080	0.428
Sex (male/female)	**0.139**	**0.024, 0.802**	**0.027**
Total years of education(years)	1.115	0.875, 1.421	0.378
Current alcohol use (at least weekly) (yes/no)	0.250	0.053, 1.182	0.080
Current Nicotine use (yes/no)	1.004	0.203, 4.961	0.996
Current Marijuana use (yes/no)	**6.056**	**1.168, 31.397**	**0.032**
Age of MDD onset (years)	**1.086**	**1.009, 1.169**	**0.028**
Length of Current MDE (months)	1.007	0.989, 1.025	0.468
Family history of mental illness (yes/no)	**5.476**	**1.156, 25.947**	**0.032**

* *Patients who had anhedonia at baseline and did not drop out at endpoint. Bold values denote statistical significance at the p < 0.05 level*.

### The Correlation Between Anhedonia and Functional Impairment

**Figure 2:**
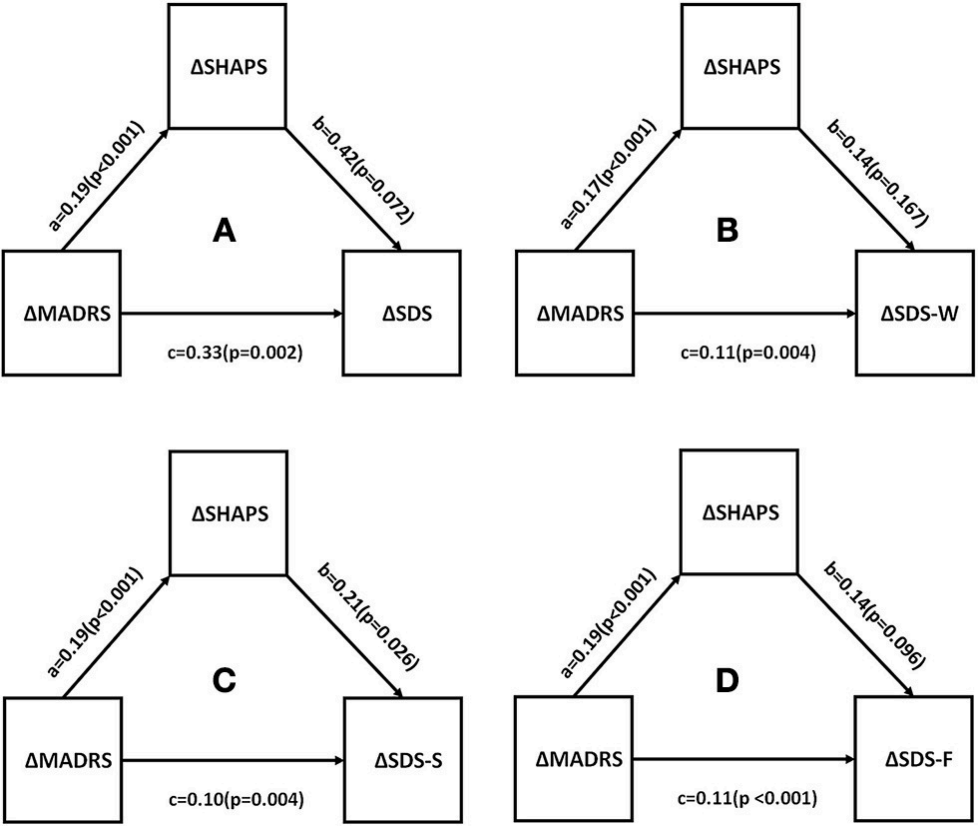
Mediation analysis to estimate indirect effects of anhedonia improvement (ΔSHAPS) in the improvement of depressive symptoms (ΔMADRS) and function (ΔSDS). a, b, and c are path coefficients. **(A)** SDS total score, **(B)** SDS- work/school; **(C)** SDS -social life; **(D)** SDS-family life or home responsibilities. MADRS, Montgomery Åsberg Depression Rating Scale; SHAPS, Snaith-Hamilton Pleasure Scale; SDS, Sheehan Disability Scale.

**Figure 3:**
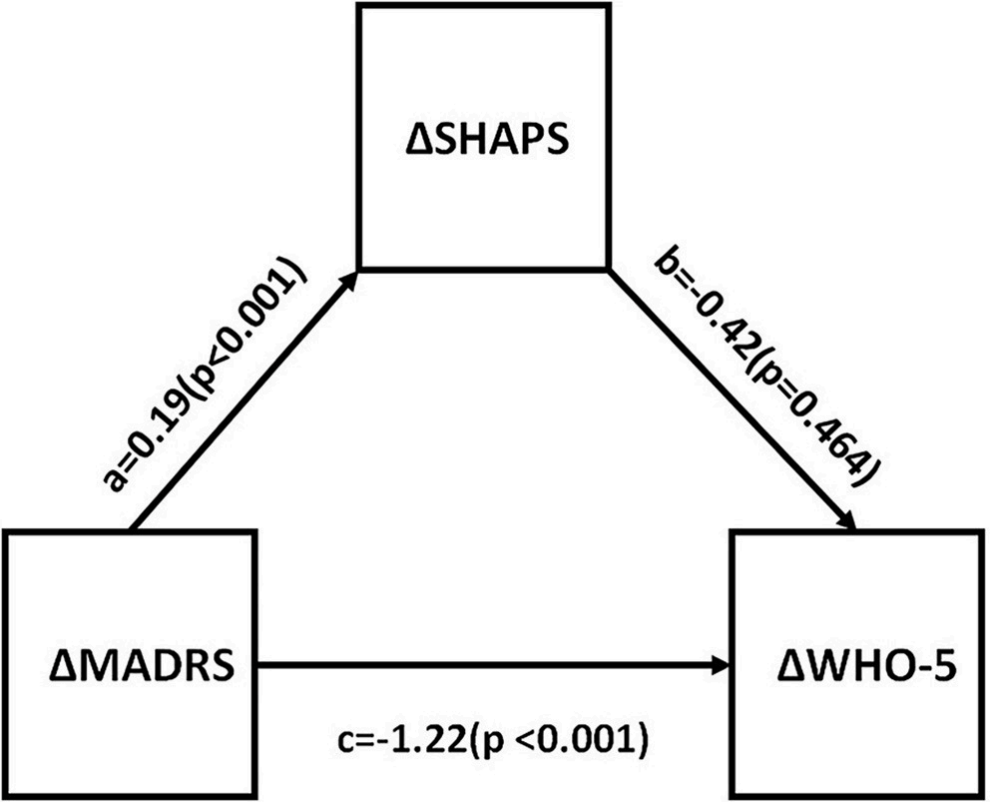
Mediation analysis to estimate indirect effects of anhedonia improvement (ΔSHAPS) in the improvement of depressive symptoms (ΔMADRS) and well-being (ΔWHO-5). a, b, and c are path coefficients. MADRS, Montgomery Åsberg Depression Rating Scale; SHAPS, Snaith-Hamilton Pleasure Scale; WHO-5, The World Health Organization- Five Well-Being Index.

Correlations between changes in depressive symptom severity, anhedonia, functional impairment, and quality of life were assessed. All measures were found to be significantly different between baseline and endpoint (all *p* < 0.05). This is shown in Table 4. Mediational analysis was used to evaluate he indirect effect of improvements in anhedonia (ΔSHAPS) on the association between changes in depressive symptom severity (ΔMADRS) and changes in function (ΔSDS) [as well as well-being (ΔWHO-5)] (Figures 2, 3). The results showed that anhedonia improvement was a strong mediator of the association between improvement in depressive symptoms and improvement in social functioning [i.e., improvement in the social life component of the SDS (ΔSDS-S)] with *p* = 0.026, and explained 39.9% of the total variance. Anhedonia improvement was not found to mediate the association between improvement in depressive symptoms and improvement in other SDS domains or WHO-5.

**Table 4:** The correlations of the endpoint changes between functional impairment, well-being and anhedonia from baseline.

**Correlations**	**SDS total score**		**SDS work**		**SDS social life**		**SDS family life**		**WHO-5**	
	***r***	***p***	***r***	***p***	***r***	***p***	***r***	***p***	***r***	***p***
MADRS total score	0.527	< 0.001	0.422	< 0.001	0.46	< 0.001	0.486	< 0.001	−0.604	< 0.001
SHAPS score	0.392	< 0.001	0.309	0.006	0.403	< 0.001	0.364	0.001	−0.336	0.002
MADRS anhedonia factor score	0.511	< 0.001	0.423	< 0.001	0.41	< 0.001	0.507	< 0.001	−0.570	< 0.001

*MADRS, Montgomery Åsberg Depression Rating Scale; SHAPS, Snaith-Hamilton Pleasure Scale; SDS, Sheehan Disability Scale; WHO-5, The World Health Organization- Five Well-Being Index*.

## Discussion

Anhedonia is subserved by a dysregulation of central nervous system reward circuits and structures (20), and is a composite symptom with two primary dimensions (i.e., motivational/appetitive and consummatory dimensions) (21, 22). The present analysis indicated that both outcome measures (i.e., SHAPS and MADRS anhedonia component) were highly correlated (with each other) and could reliably assess anhedonia symptoms. We evaluated anhedonia as a continuous and categorical measure. Definitions for response and remission with the SHAPS have been reported elsewhere (19). Herein, we observed that 70.5% of our sample met SHAPS-defined criteria for clinically significant anhedonia symptoms at baseline, underscoring the high prevalence of this disturbance. After 8 weeks of flexibly dosed vortioxetine, it was observed that 56.9% of participants met SHAPS-defined response criteria.

Previous studies have reported anti-anhedonia effects in adults with MDD treated with select antidepressants including, but not limited to agomelatine, bupropion, venlafaxine, fluoxetine, amitifadine, levomilnacipran, escitalopram, and ketamine (22–26). Moreover, some antidepressants (e.g., SSRIs) have been shown to help treat emotional blunting in MDD, which phenotypically overlaps with anhedonia in some subjects (27). The results of the present study indicate that vortioxetine may also be an effective treatment of anhedonia in MDD. Herein, we observed a significant benefit of vortioxetine on anhedonia, as measured by the SHAPS and MADRS anhedonia factor. We observed that the improvement in anhedonia with vortioxetine treatment was significantly correlated with improvement in function and quality of life. Moreover, we observed a significant mediational effect of improvement in anhedonia on social functioning, which was independent of the effect of vortioxetine treatment on total depression symptom severity.

We also identified four variables that may affect remission of anhedonia in patients treated with vortioxetine (i.e., sex, marijuana use, age of MDD onset, and family history of mental illness). We found that female subjects with MDD treated with vortioxetine were more likely to achieve remission status (i.e., SHAPS total score < 3). In other words, the anti-anhedonia effects with vortioxetine may be more pronounced in women with MDD. Although sex differences have been reported in MDD with respect to phenomenology, comorbidity, illness trajectory, and response to treatment, it is not known whether sex differences exist with respect to the likelihood of exhibiting an anti-anhedonia effect with antidepressant treatment. It is also noteworthy that subjects in our study who reported regular current use of marijuana were less likely to exhibit an anti-anhedonia effect with treatment. The foregoing observation requires replication and comports with separate lines of evidence indicating that recreational marijuana utilization exerts both anhedonia-promoting and anti-cognitive effects in users (28, 29). Furthermore, we further observed that individuals with MDD reporting with a family history positive for psychiatric illness were less likely to exhibit an improvement in anhedonia. It could be conjectured that the increased loading of psychopathology in families represents a more complex, less treatment responsive phenotype and/or ongoing environmental stressors as a general non-response predictor (30).

Improvements in overall function and quality of life have been prioritized as a primary therapeutic objective with respect to treatment of patients with MDD (31). Relatively few antidepressants have been evaluated with respect to their primary effect on psychosocial function, workplace performance, and return work (32). Several lines of research indicate that vortioxetine is capable of improving psychosocial function, as assessed by self-report and/or performance based measures (33–35). The results from the present study replicate previous studies showing that vortioxetine improves both psychosocial function and quality of life. We extend further existing knowledge by observing that improvements in function and quality of life were mediated by improvement in measures of anhedonia. Final, the foregoing observations with vortioxetine comports with previous research findings demonstrating that improvement in anhedonia mediates the association between improvements in depressive symptoms and improvements psychosocial functioning (19).

There are methodological limitations that affect inferences and interpretations of our data analyses. First, our analyses were done *post-hoc*. Second, evaluating the effect of vortioxetine on anhedonia (and the mediational effect of anhedonia on function and quality of life) was not the primary aim of the study. Third, our study was open-label and not placebo-controlled, increasing the likelihood of expectancy affecting our outcomes of interest. In addition, we did not exclude the patients received medications could effect anhedonia at screening or measure the anhedonia status during the tapering off period. Last, our analysis did not include a more rigorous performance-based measure of reward/motivation (e.g., EEFRT) (36). Notwithstanding, our analyses were meant to be more hypothesis-generating and to provide pilot data supporting a larger rigorous study evaluating determinants of improved functional outcomes with vortioxetine.

In summary, vortioxetine improved measures of anhedonia, which significantly correlated with improvements in function. Moreover, the effect of improvement in anhedonia on patient-reported outcomes (i.e., social functioning) was independent of the overall improvement in depressive symptoms. Our results, if replicated, would indicate that measures of reward/motivation, along with cognitive disturbance are critical determinants of health outcomes in patients with MDD.

## Ethics Statement

The study has been reviewed and approved by an independent Institutional Review Board that is accredited by the Association for the Accreditation of Human Research Protection.

## Author Contributions

RSM was responsible for designing the study. BC was responsible for analyzing data and writing the original manuscript. CP, MS, HZ, MI, and RBM collected the research data. RSM, CP, MS, MI, YL, and LP revised the manuscript. All authors have read and approved the final version of this article.

### Conflict of Interest Statement

RSM is a consultant for, and/or has received research support from Lundbeck, Janssen, Shire, Purdue, Pfizer, Otsuka, Allergan, Takeda, Neurocrine, Sunovion, Stanley Medical Research Institute, and CIHR/GACD/Chinese National Natural Research Foundation. The remaining authors declare that the research was conducted in the absence of any commercial or financial relationships that could be construed as a potential conflict of interest.
