# Supplementary material for: The Efficacy of Vortioxetine on Anhedonia in Patients With Major Depressive Disorder
Source: Front Psychiatry. 2019 Jan 31;10:17. doi: 10.3389/fpsyt.2019.00017 (PMC6365446; doi:10.3389/fpsyt.2019.00017)
Supplement: Supplementary file 1 [file Table_1.docx]

**Supplemental Table 1. The changes of SHAPS and MADRS anhedonia factor scores with treatment of vortioxetine in patients with SHAPS score > 3 at baseline.**

| Variables | **Baseline (n=67)** |  | **Week 2 (n=66)** | | | |  | **Week 8 (n=58)** | | | |
| --- | --- | --- | --- | --- | --- | --- | --- | --- | --- | --- | --- |
|  | M±SD |  | M±SD | *Δbaseline (95% CI)* | *z* | *p* |  | M±SD | *Δbaseline (95% CI)* | *z* | *p* |
| **SHAPS score** | 8.1±2.7 |  | 6.7±4.3 | -1.4 (-2.3, -0.5) | -3.04 | 0.002*** |  | 4.3±4.1 | -3.8 (-4.8, -2.9) | -8.03 | <0.0001*** |
| **MADRS anhedonia factor** | 19.0±3.5 |  | 15.2±5.3 | -3.9 (-5.1, -2.6) | -5.98 | <0.0001*** |  | 12.3±5.9 | -6.9 (-8.2, -5.5) | -10.10 | <0.0001*** |

* indicates significant differences from baseline.
